# Ketogenic diet for mitochondrial disease: a systematic review on efficacy and safety

**DOI:** 10.1186/s13023-021-01927-w

**Published:** 2021-07-03

**Authors:** Heidi Zweers, Annemiek M. J. van Wegberg, Mirian C. H. Janssen, Saskia B. Wortmann

**Affiliations:** 1 Department of Gastroenterology and Hepatology - Dietetics, Radboudumc, Postbus 9101, 6500 HB Nijmegen, The Netherlands; 2 Radboud Center for Mitochondrial Medicine (RCMM), Amalia Children’s Hospital, Radboudumc, Nijmegen, The Netherlands; 3 University Children’s Hospital, Paracelsus Medical University, Salzburg, Austria; 4 Department of Internal Medicine, Radboudumc, Nijmegen, The Netherlands

**Keywords:** Epilepsy, Complex I, Treatment, Management, Mitochondrial myopathy, OXPHOS, Mitochondrial DNA deletion, Adverse event, Modified Atkins diet, High fat diet

## Abstract

**Background:**

No curative therapy for mitochondrial disease (MD) exists, prioritizing supportive treatment for symptom relief. In animal and cell models ketones decrease oxidative stress, increase antioxidants and scavenge free radicals, putting ketogenic diets (KDs) on the list of management options for MD. Furthermore, KDs are well-known, safe and effective treatments for epilepsy, a frequent symptom of MD. This systematic review evaluates efficacy and safety of KD for MD.

**Methods:**

We searched Pubmed, Cochrane, Embase and Cinahl (November 2020) with search terms linked to MD and KD. From the identified records, we excluded studies on Pyruvate Dehydrogenase Complex deficiency. From these eligible reports, cases without a genetically confirmed diagnosis and cases without sufficient data on KD and clinical course were excluded. The remaining studies were included in the qualitative analysis.

**Results:**

Only 20 cases (14 pediatric) from the 694 papers identified met the inclusion criteria (one controlled trial (n = 5), 15 case reports). KD led to seizure control in 7 out of 8 cases and improved muscular symptoms in 3 of 10 individuals. In 4 of 20 cases KD reversed the clinical phenotype (e.g. cardiomyopathy, movement disorder). In 5 adults with mitochondrial DNA deletion(s) related myopathy rhabdomyolysis led to cessation of KD. Three individuals with *POLG* mutations died while being on KD, however, their survival was not different compared to individuals with *POLG* mutations without KD.

**Conclusion:**

Data on efficacy and safety of KD for MD is too scarce for general recommendations. KD should be considered in individuals with MD and therapy refractory epilepsy, while KD is contraindicated in mitochondrial DNA deletion(s) related myopathy. When considering KD for MD the high rate of adverse effects should be taken into account, but also spectacular improvements in individual cases. KD is a highly individual management option in this fragile patient group and requires an experienced team. To increase knowledge on this—individually—promising management option more (prospective) studies using adequate outcome measures are crucial.

**Supplementary Information:**

The online version contains supplementary material available at 10.1186/s13023-021-01927-w.

## Introduction

Mitochondrial diseases (MDs) are a heterogenous group of inborn metabolic diseases caused by defects in the genes encoding mitochondrial proteins that are required for ATP production from oxidation of substrates via the tricarboxylic acid cycle and the oxidative phosphorylation (OXPHOS). Underlying pathogenic variants can be found in nuclear or mitochondrial DNA (mtDNA) [1, 2]. Currently more than 295 different disorders are known [2]. Virtual all the extremely heterogeneous symptoms can occur with onset at all ages, but typically tissues with high energy requirements like skeletal/heart muscle and brain are mainly affected. Currently no curative treatment is available, making the supportive management for symptom relief priority.

Given the key role of mitochondria in energy metabolism and the importance of vitamins and co-factors for proper mitochondrial function, nutritional interventions are an integral component of daily management [3–7]. Moreover, nutritional interventions focusing on nutritional status or gastro-intestinal complaints have been shown effective [3, 4].

A ketogenic diet (KD) is a low-carbohydrate high-fat diet that shifts metabolism towards β-oxidation and ketone body production. Three kind of KDs are defined. The classic KD uses grams of fat: grams of carbohydrate plus protein-ratio (e.g. 4:1 or 3:1) in every meal. Fat can be (mainly) given as medium-chain triglyceride (MCT), this subtype is called MCT-KD. As MCT-fats are converted easier into ketones than longer chain fatty acids, ketosis can be achieved more easily and more carbohydrates can be consumed. In contrast, the modified Atkins diet (MAD) only restricts carbohydrates (10–20 g per day) without restricting the amount of fat and protein [8–10].

KDs have been proven successful in the treatment of intractable epilepsy and are generally well tolerated and safe [9, 11, 12]. It is thought that KDs exert their positive effect (among others) via stimulation of mitochondrial biogenesis, improvement of mitochondrial function and decrease of oxidative stress [13–16] and therefore have been implemented in some cases with MD and epilepsy [12, 17]. There are also studies suggesting a potential beneficial effect of KD in MD, besides reducing seizures [14, 18]. However, this was mainly studied in patient derived fibroblasts and animal models [8, 14, 19–23]. Of note, while it was previously assumed that the liver provides ketone bodies to the brain, astrocytes itself have shown to be ketogenic cells. This astrocyte ketogenesis might control the survival/death decision of neural cells at least twofold. By scavenging non-esterified fatty acids the ketogenic pathway could prevent the detrimental actions of these metabolites and their derivatives (e.g. ceramide) on brain structure and function. Further, by acting directly as pro-survival metabolites, the ketone bodies may preserve neuronal synaptic function and structural stability [24].

**Fig. 1:**
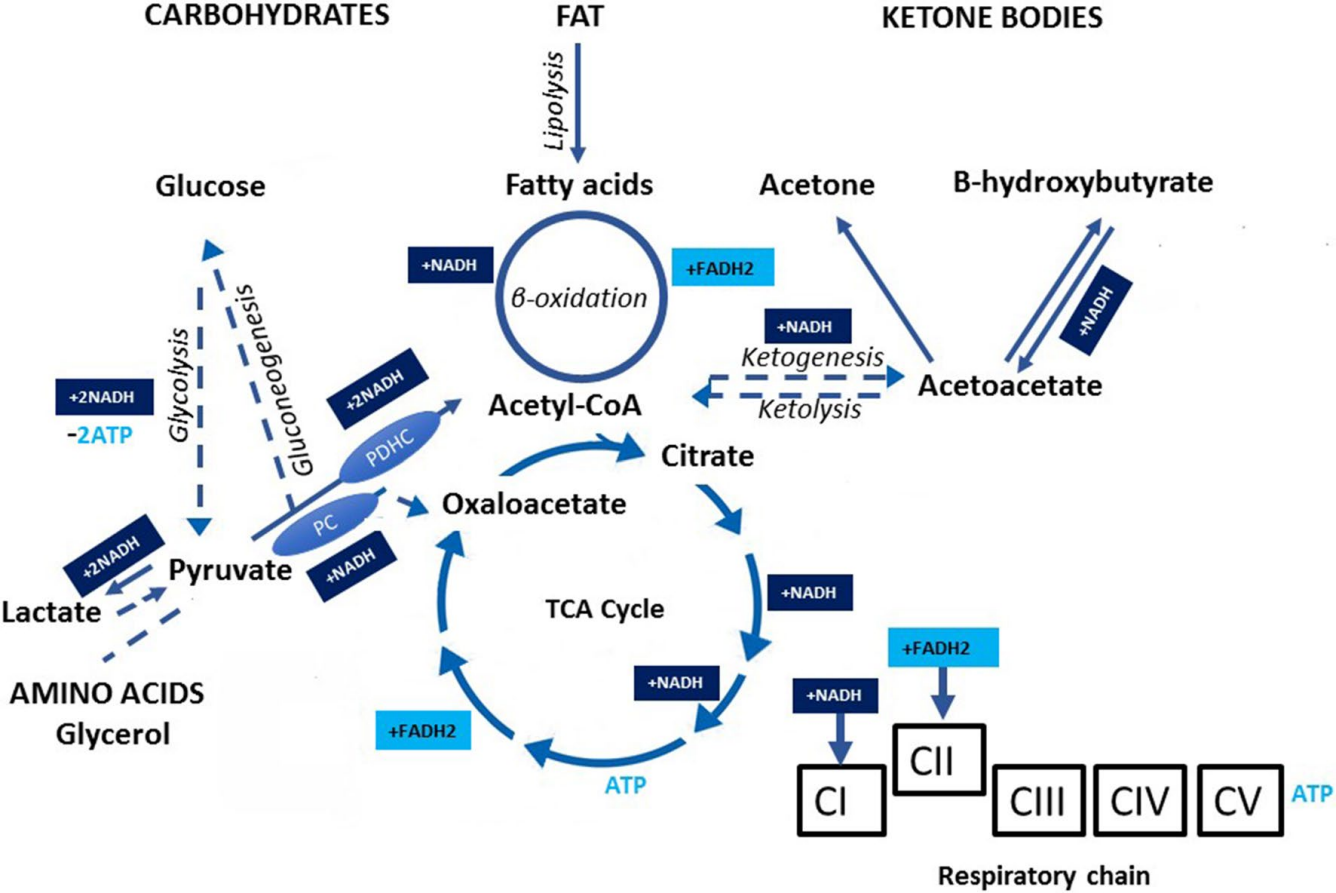
Metabolic pathways of carbohydrates, fat and ketone bodies in energy metabolism. *ATP* adenosine triphosphate, *C* respiratory chain complex, *PDHC* pyruvate dehydrogenase complex, *PC* pyruvate carboxylase, *TCA Cycle* Tricarboxylic acid cycle also called citric acid cycle

When a diet provides only small amounts of glucose, hormones as glucagon inhibit glycolysis and stimulate ketogenesis. These ketone bodies can only be produced in the liver and in astrocytes, and provide the mitochondrial OXPHOS with a substrate for energy production. The fatty acid pathway provides 5.7 times more flavin adenine dinucleotide (FADH2) than the glycose pathway and therefore fat has a potential benefit over carbohydrates as an energy substrate in human complex 1 deficiency [25]. However nicotinamide-adenine-dinucleotide (NADH) is still formed from all substrates and complex 1 is never completely bypassed (Fig. 1).

Pyruvate dehydrogenase complex (PDHC) deficiency hampers the conversion of pyruvate to acetyl-CoA and KDs are the pathomechanism based therapy as ketones, converted to acetyl-CoA, bypass the PDHC [26]. Pyruvate Carboxylase deficiency is a contraindication for KD as gluconeogenesis is impaired and affected individuals depend on nutritional glucose.

Taken together, KDs are an interesting management option for MD that needs further evaluation. We here perform a systematic literature review to assess efficacy and safety of KD for MD.

## Methods

### Search strategy

This systematic review was conducted according to the Preferred Reporting Items for Systematic Reviews and Meta-Analyses (PRISMA) guidelines [27]. We identified relevant studies using medical subject headings (MeSH) and text words related to KD and MD (see Additional file 1). The databases searched were: Pubmed, Cochrane, Embase and Cinahl (November 2020) without any search limits. The search strategy for Pubmed was generated together with a specialist librarian and accordingly amended for the other databases.

### Study selection

Two authors (HZ, AvW) independently screened and selected the papers using Rayyan® [28]. Eligibility criteria were: cases with MD using a KD and English language. The same authors reviewed full-texts of these selected papers independently, according to exclusion criteria. Disagreements were resolved by consensus and discussion with a third author (SBW). Exclusion criteria were: (i) cases without genetically proven MD [1, 2], (ii) cases with PDHC-deficiency, (iii) cases not on KD or without details of the KD composition and (iv) cases without data of effect on clinical phenotype before and under treatment.

KD was defined as any dietary manipulation of fat, carbohydrate and protein aiming to achieve ketosis and included the ‘classic’ KD, MCT-KD or MAD [8–10]. High fat diets including the low glycaemic index diet are not likely to achieve ketosis and therefore cases treated with these diets were excluded.

Reference lists were reviewed for additional publications.

### Outcome measures

The primary outcome was the effect of KD on clinical phenotypes (epilepsy, muscle involvement, tonus dysregulation (muscular hyper- or hypotonia), movement disorders, developmental delay and intellectual disability (DD/ID), other individual signs and symptoms) and the occurrence of adverse events (AEs). The secondary outcome was defined as the effect of KD on MRI findings and laboratory values (e.g. lactic acidosis, liver function test).

### Data extraction and quality appraisal

Two authors (HZ, AvW) extracted data and checked the data for completeness. A third author (SBW) checked all articles again to ensure correct interpretation of data. Discrepancies were resolved through discussion and consensus.

We used The Oxford Levels of Evidence 2 [29] scoring to assess study quality as well as the The Risk Of Bias In Non-randomized Studies—of Interventions (ROBINS-I) assessment tool [30]. No studies were suitable for pooling of the results and therefore a narrative analysis is presented.

## Results

**Fig. 2:**
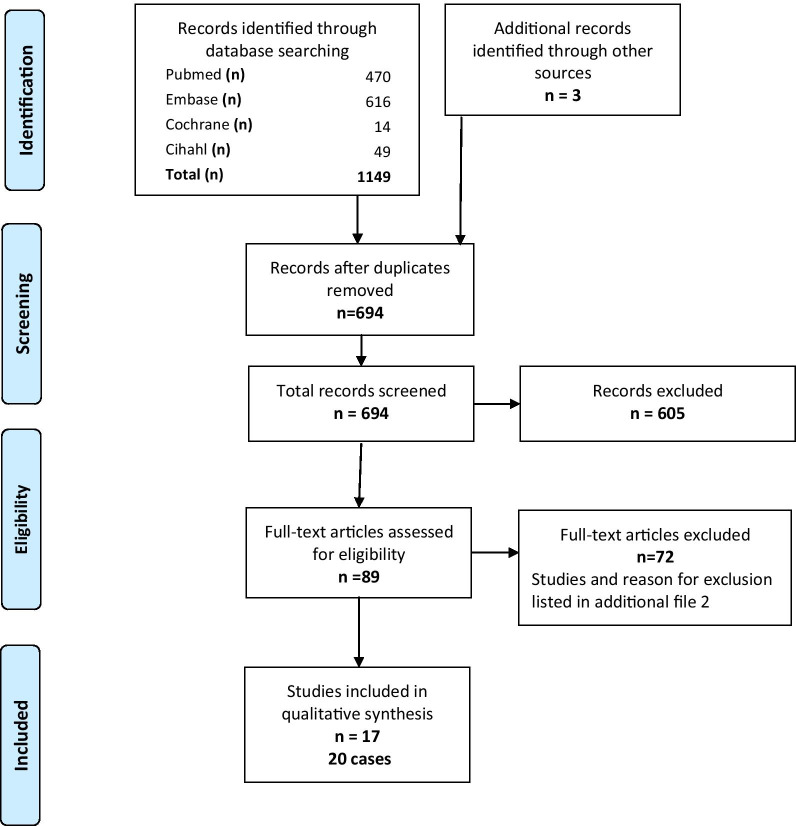
PRISMA flowchart. This figure detailing the search strategy

**Fig. 3:**
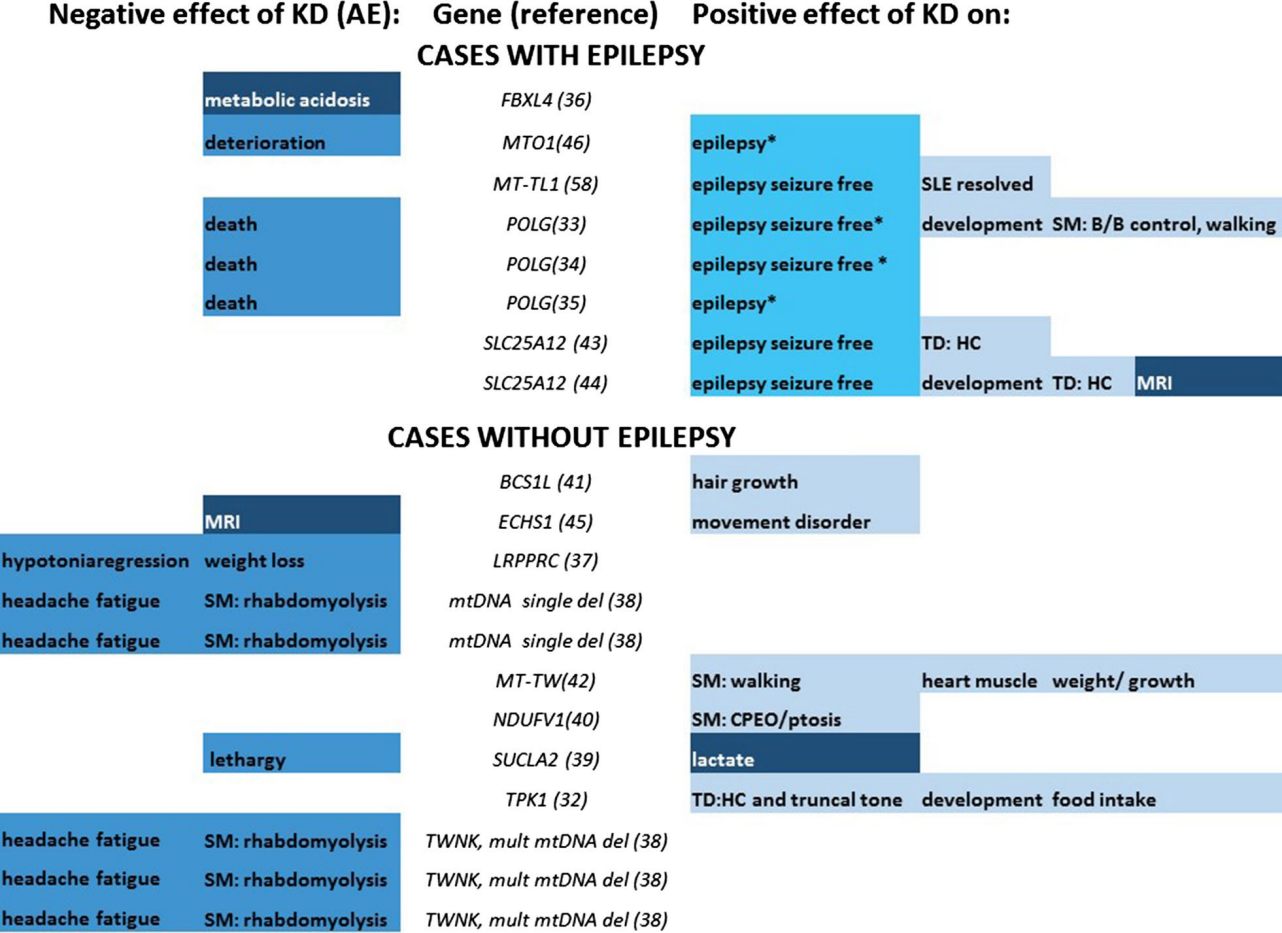
Summary of positive and negative effects of ketogenic diet in 20 cases with genetically proven mitochondrial disease. This figure visualises the negative effects and adverse events on the left and the positive effects (on the right) of ketogenic diet in 20 cases with genetically proven mitochondrial disease. *temporary effect **cases with reported treatment-withdrawal effect. *B/B* bladder and bowel, *CPEO* chronic progressive external ophthalmoplegia, *del* deletions, *HC* head control, *mult* multiple deletions in mitochondrial DNA, *MRI* magnetic resonance imaging, *mtDNA* mitochondrial DNA, *SLE* stroke like episodes, *SM* skeletal muscle, *TD* tonus dysregulation

The search strategy yielded 1149 abstracts (PRISMA flowchart, Fig. 2 [31]) of which 17 papers reporting 20 cases were included in the detailed analysis. All data are summarized in Table 1 and Fig. 3. Of note, (multi)vitamins and other food supplements were reported in many of the included cases (see Table 1 for details). With exception of one case (*TPK1* [32]) clinically not responding to thiamine supplementations, none of the reported vitamins or co-factors were pathomechanism based treatment options and therefore were not taken into account in our analysis.

**Table 1:** Details on genotype, phenotype, diet intervention, safety and efficacy of all 20 cases split into cases with and without epilepsy

Author and year (reference)	Involved Gene	MD subgroup	Age start KD (y)	Gender	Composition KD	Ketosis	KD duration (m)	Reason stop KD	Supplements	Development*	Muscles and movement*	Other signs and symptoms*	Adverse events	Positive effects on
*Cases with epilepsy*														
Köse 2020 [36]	*FBXL4 (AR)*	Unclear function	0.8	M	NR	+	0.2 (5d)	AE	B, CoQ, R, T	DD	TD	Visual impairment, bowel dysmotility bleeding, dysphagia, FP	Metabolic acidosis	NR
O'Byrne 2018 [46]	*MTO1 (AR)*	DNA, RNA and protein synthesis/RNA metabolism	7	F	CKD 4,75:1–2:1	NR	> 108	NA	CA, CoQ, D, E, R, T	DD	MW, TD, ataxia	behavioural issues	Sudden deterioration during flu-like illness (visual impairment, ptosis, generalised weakness)	E (temporary seizure reduction for 4 y)
Steriade, 2014 [58]	*MT-TL1 (maternal)*	DNA, RNA and protein synthesis/tRNA	22	F	MAD	NR	> 12	NA	ALA, BC, CA,CoQ, CR, D, E, FA	NR	NR	Migraine, stroke like episodes		E (seizure free for > 1 y), stroke like episodes (resolved)
Joshi 2009 [33]	*POLG (AR)*	DNA, RNA and protein synthesis/Replication	4.6	F	CKD 4:1	NR	9	AE	NR	DD	MW, gait ataxia	Visual impairment	Death (at age 66 m)	E (temporary seizure free for 7 m), muscle (bladder/bowel control regained, walking with assistance regained), development (ability to speak)
Spiegler 2011 [34]	*POLG (AR)*	DNA, RNA and protein synthesis/Replication	3.6	F	CKD	NR	3	AE	NR	DD	ataxia	Bowel obstruction, dysphagia	Death (at age 46 m)	E (temporary seizure free for a few w)
Koessler 2021 [35]	*POLG (AR)*	DNA, RNA and protein synthesis/Replication	16	F	CKD 4:1	+	3	AE	CoQ, R, T	NR	NR	Migraine	Death (at age 16 y)	E (temporary improvement of status epilepticus for 3 w)
Pfeiffer 2020 [43]	*SLC25A12 (AR)*	Substrate/Carrier	1.8	M	CKD 4:1	NR	> 4	NA	NR	DD	TD	NR		E (seizure free for > 4 m, TD (improved head and neck control)
Dahlin 2015 [44], Wibon 2009 [59]	*SLC25A12 (AR)*	Substrate/Carrier	6	F	CKD 3–4:1	+	> 20	NA	NR	DD	TD	NR		E (seizure free for > 20 m), TD (improved head and neck control) development, (psychomotor, social interaction), MRI
*Cases without epilepsy*														
Della-Marina, 2020 [41]	*BCS1L (AR)*	Assembly, complex II	7	F	MAD	+	4	PD	NR	NR	NR	Hearing loss, alopecia, sparse, brittle hair		**Hair growth**
Illsinger, 2020 [45]	*ECHS1 (AR)*	Inhibitors	4	F	MAD 1:1	NR	> 60	NA	B, T	NR	dystonia	NR	Worsening of MRI	**Movement disorder (resolved)**
Kotecha, 2019 [37]	*LRPPRC (AR)*	DNA, RNA and protein synthesis/RNA metabolism	0 (7d)	F	CKD	+	3.3	AE	NR	NR		Respiratory distress	Progressive hypotonia and regression, weight loss	NR
Ahola, 2016 [38]	*mtDNA single del (maternal)*	mtDNA single deletion	62	F	MAD	+	0.1 (4d)	AE	NR	NR	MW, EI, ptosis	NR	RM, headache, tiredness	NR
Ahola, 2016 [38]	*mtDNA single del (maternal)*	mtDNA single deletion	36	F	MAD	+	0.3 (8d)	AE	NR	NR	MW, EI, ptosis	NR	RM, headache, tiredness	NR
Deberles, 2020 [42]	*MT-TW* *(maternal)*	t-RNA	3	F	CKD 3:1	+	> 96	NA	C, CA, E, R, T	DD	MW, CM	FTT		Muscle (regained walking, improved limb muscle strength), cardiomyopathy (resolved), weight gain, growth
Laugel 2007 [40]	*NDUFV1 (AR)*	OXPHOS enzymes/complex I	0.8	M	CKD 3:1	+	24	PD	CoQ, R	DD	MW, TD, ptosis, ataxia, pyramidal signs	Vomiting, hyperpnea, strabismus		**CPEO, ptosis (resolved)**
Huang 2017 [39]	*SUCLA2 (AR)*	DNA, RNA and protein synthesis/Nucleotides	1.3	M	CKD 3:1	+	5	AE	C, CA, CoQ, E, R, T	DD	MW, TD ptosis, hyper-reflexia	Hearing loss, FTT, FP, GER, constipation	Severe lethargy	Lactate (normalised)
Fraser 2014 [32]	*TPK1 (AR)*	Cofactors/Thiamine	1.7	M	CKD 3:1	+	> 9	NA	ALA, B, N, T	DD	TD	FP		TD (improved head and neck control, truncal tone stability), development (increased verbal response and social interaction), food intake
Ahola, 2016 [38]	*TWNK (AR), mult del (maternal)*	DNA, RNA and protein synthesis/Replication	54	M	MAD	+	0.3 (9d)	AE	NR	NR	MW, EI, ptosis	NR	RM, headache, tiredness	NR
Ahola, 2016 [38]	*TWNK (AR), mult del (maternal)*	DNA, RNA and protein synthesis/Replication	52	M	MAD	+	0.3 (8d)	AE	NR	NR	MW, EI, ptosis	NR	RM, headache, tiredness	NR
Ahola, 2016 [38]	*TWNK (AR), mult del (maternal)*	DNA, RNA and protein synthesis/Replication	40	m	MAD	+	0.4 (11d)	AE	NR	NR	MW, EI, ptosis	NR	RM, headache, tiredness	NR

AE = adverse event, ALA = alpha lipoic acid, AR = autosomal recessive, B = biotin, BC = vitamin B complex,C = Vitamin C, CA = carnitine, CR = creatine,CKD = classical ketogenic diet, CM = Cardio Myopathy, CoQ = idebenone or coenzyme Q10, CPEO = chronic progressive external ophthalmoplegia, d = days, DD = developmental delay, E = epilepsy, F = female, EI = exercise intolerance, FA = folic acid, FFT = failure to thrive, FP = feeding problems, GER = gastroesophageal reflux, ID = intellectual disability, KD = ketogenic diet, m = months M = male, MAD = modified atkins diet, MRI = magnetic resonance imaging, MW = muscle weakness, N = niacin, NA = not applicable, NR = not reported, PD = parental decision, R = riboflavin, RM = rhabdomyolysis, T = thiamin, TD = tonus dysregulation, w = weeks, y = years, * = clinical findings before start KD. in bold: cases with reported treatment-withdrawal effect

### Case characteristics

Of the 20 cases (12 female), 16 had a nuclear DNA and 4 a mtDNA related mutation. Of note 3 of the nuclear variants (*TWNK)* lead to multiple mtDNA deletions. The ages at start of KD ranged between 0 and 62 years (1 neonate (aged 7 days), 2 infants (9 and 10 months), 11 children (1.3–16 years), 6 adults (aged 22–62 years)). Eight individuals (8/20), were described as having epilepsy. In 10/20 individuals muscle involvement (cardiomyopathy, muscle weakness, exercise intolerance, ptosis) was reported, 7/20 were described having tonus dysregulation, and for 5/20 cases movement disorder(s) (ataxia, dystonia) were reported. In 10/20 cases DD/ID was reported. Other reported symptoms were visual problems, respiratory distress, headaches/migraine, failure to thrive, feeding problems, gastro-intestinal problems, alopecia and hearing loss.

### Study quality

The general study quality was low, with 15 case reports (category 4) and one controlled trial with 5 adult participants (category 3b). The risk of bias in the trial was scored as low.

### Interventions

Eleven cases followed a classical KD, 8 cases a MAD. For one case the composition of the KD was not detailed, however, this case was included as achievement of ketosis was documented. For a total of 14 cases achievement of ketosis was reported. The total diet duration of all 20 cases together was > 22 patient years, the median diet duration was 4 months (range 4 days -9 years). The main reasons for discontinuation of the diet (n = 12) were death (n = 3) [33–35] and other AEs (n = 7) [36–39]. In one case the daily constraints [40] and in one case the child’s craving for carbohydrates and lack of improvement of hearing [41] lead to cessation after 2 years and 4 months, respectively.

### Primary outcomes

#### Effect of KD on epilepsy

In 7/8 cases with uncontrolled epilepsy seizure control was achieved with KD (5 seizure free, 1 reduction of seizures and 1 “stabilisation” of status epilepticus). In 4 of these cases the effect was only temporary (lasting a few weeks to 7 months). In one case no positive effect of the KD on epilepsy was reported, and the KD was stopped after 5 days due to metabolic acidosis.

#### Effect of KD on other clinical signs and symptoms

##### Skeletal and heart muscle

In 3/10 cases with muscle involvement KD had a positive effect. This was the return of bladder and bowel control, and the ability to walk with assistance [33], and the complete resolve of chronic progressive ophthalmoplegia (CPEO) and ptosis [40]. In the third case, the 3-year-old individual regained walking abilities with improved lower limb muscle strength after one year of KD and normalisation of septum thickness of hypertrophic cardiomyopathy after 3 years of KD. This effect was sustained at the last follow up at age 11 years (total 8 years of KD) [42]. A negative effect was observed in 6/10 individuals. Five of these six individuals experienced rhabdomyolysis, headache and fatigue leading to cessation of KD after 4 to 11 days [38] and in one case progressive muscular hypotonia with swallowing difficulties was reported going along with a weight loss below the 3^rd^ percentile [37].

##### Tonus dysregulation

Tonus dysregulation was reported to improve in 3/7 cases, while in one case progressive muscular hypotonia was only reported after the KD was started [37]. In all 3 cases head control improved [32, 43, 44] and one case additionally showed improved stability of truncal tone enabling him to stand with support and to sit independently [32].

##### Movement disorder

In one case the paroxysmal ophisthotonic dystonia completely resolved for more than 5 years of follow-up [45]. In one individual the ataxia did not improve [46], in the other 3 cases no further details were provided [33, 34, 40].

##### Developmental delay/Intellectual disability

In 3/10 cases with DD/ID a positive effect of KD was reported (e.g. increased verbal response and abilities, social interaction, improved memory) however no data of formal psychological or developmental testing were presented [32, 33, 44]. The other publications did not provide further details.

##### Other

An improved oral food intake [32], improved weight gain and growth [42] and hair growth in an individual with alopecia [41] were reported in one case each.

##### Treatment-withdrawal effect

In 3 cases a treatment withdrawal effect was demonstrated. In the case where KD had resulted in hair growth, this was lost again 6 months after cessation of KD [41]. In the individual where KD led to dissapearance of the dystonic-opisthotonic episodes within 5 days for 5 months, these reoccured within a few days when the KD was stopped, and dissapeared again 4 weeks after reintroducing KD [45]. In the case where CPEO and ptosis resolved within days and remained absent for 2 years on KD, the ptosis partially reoccured upon “relaxation" of the diet [40].

#### Adverse events

In 13/20 individuals AEs were reported (Table 1). In one study [38] all 5 adult participants with mitochondrial myopathy (2 mtDNA single deletion, 3 *TWNK*/multiple mtDNA deletions*)* stopped MAD within 4–11 days because of rhabdomyolysis, headache and tiredness.

Three individuals, all with *POLG*-related Alpers syndrome, died during KD (at the ages of 46 months [34], 66 months [33] and 16 years [35]) of respiratory failure (n = 2) or paralytic bowel obstruction (n = 1). This was 3 months (n = 2) [34, 35] and 35 months [33] respectively, after presentation.

Other AEs that lead to immediate cessation of the KD were severe lethargy, which occurred after 5 months of KD in one case [39] and lactic acidosis (after 5 days of KD) in one other case [36]. Of note, two individuals in two reports remained on KD despite AEs. In one case because of successful seizure reduction despite a sudden deterioration of visual acuity, ptosis and general weakness after 6 months of KD [46] and in one case the resolve of the movement disorder outweighed the worsening seen on MRI [45]. In one individual [37] who suffered from weight loss, regression and hypotonia the KD was continued for 3.3 months and then weaned to a more conventional feeding regime.

### Secondary outcomes

From the 12 cases with reported MRI abnormalities for only 2 cases MRI details were provided after KD initiation. In one case a resumed myelination during KD was observed [44], while in the second the MRI worsened [45]. Interestingly in the latter case, the movement disorder had completely resolved and the patient was reported to develop age-adequately. In 7 cases lactic acidosis was reported which normalised in one [39], worsened in one [36] and did not change in 2 cases [37, 40]. For the remaining 3 cases no details were reported [42, 43, 46]. Two individuals with *POLG* mutations had mildly elevated liver function tests before KD initiation [33, 34]. In one case these remained elevated [33], in the other case no details were provided [34]. In one individual a transient, four day long, increase in liver function tests occurred [35].

## Discussion

Despite identifying 694 studies using our search strategy, only 20 cases (one controlled trial (n = 5) and 15 case reports) were of sufficient quality for detailed analysis. These data are too scarce to draw firm conclusions regarding efficacy and safety of KD. Future reports on KD for MDs must present a minimum of “common data elements” (in line with the data shown in Table 1) describing the composition of KD as well as the clinical effect including adverse events.

### KD is effective for seizure control in MD

KD was highly effective and led to seizure control of therapy refractory seizures in 7 of 8 MD cases, at least temporarily. KD was stopped only after 5 days in the 8th case and it remains elusive if adaptation of the KD would have overcome the occurring lactic acidosis and would have led to seizure control. The number of cases does not allow comparing with the efficacy of KD for intractable seizures of other causes (up to 55%/25% becoming seizure free on 4:1 classical KD/MAD [9]).

### KD might be effective for the treatment of other signs and symptoms of MD in individual cases, but is contraindicated in mtDNA deletion(s) related myopathy

In 12/20 cases KD was initiated for other indications than epilepsy. In 5 adults with mitochondrial myopathy KD was stopped due to AEs in all participants [38]. However, a potential long term benefit cannot be excluded, as the authors report a slight improvement on muscle strength and in 6-min walking test in three of four patients after 2.5 years of follow up after cessation of KD.

In the remaining 7 pediatric cases, 5 improved clinically [32, 40–42, 45], mainly concerning muscle symptoms. Especially the well reported treatment-withdrawal effect in three cases (start/stop hair growth, resolving/reoccurring ptosis or movement disorder) illustrates the potential for KD in individual management of MD. Of note, in one case hypertrophic cardiomyopathy was completely resolved on KD and sustained without any additional medication [42]. The authors discuss that ketone bodies may have modulated cardiac metabolism. This is in line with the data suggesting that in heart failure due to metabolic dysfunction fatty acids allow for sufficient energy production while carbohydrates may contribute to declining contractile function. A role for ketones both in signalling as well as an energy source is suspected to underlie this [47].

### Safety aspects of KD for MD

AEs occurred 65% of MD cases during KD. This percentage is comparable with studies on KD for PDHC deficiency (13/19 = 68%) [26] or epilepsy with mitochondrial dysfunction (22/34 = 65%) [12, 17]. AEs of KD reported in literature for refractory epilepsy are mainly gastrointestinal complaints rarely leading to discontinuing of the diet, but also lethargy and acidosis have been reported [9, 11].

The 3 children with MD that died (aged 66–192 months) while being on KD all had *POLG-*related Alpers disease, an early lethal disorder with a median age at death of 16 (range 1–181) months [48]. Hence, their age of death is comparable. Moreover, the median survival after presentation is reported to be 5 (0.5–181) months without KD [48], and was 3 (n = 2) and 35 months, in the cases with KD reviewed here.

Hence, from these limited data it seems unlikely that KD negatively influenced mortality, but is in line with the natural disease course of childhood onset MD.

### Practical recommendations

The current guidelines on KD list complex I deficiency as a condition for which KD has been shown reportedly more beneficial when compared to the average response to KD in refractory epilepsy [11, 12, 49] in general. This pathomechanism approach assumes that fatty acids compared to carbohydrates produce more FADH that can enter complex II [12, 18, 49, 50] and hence allows (partial) bypassing of complex I. However, as outlined in the introduction (Fig. 1), NADH is still formed from all substrates and complex I is never completely bypassed.

Our study did solely include cases with known genetic background as increasing knowledge from next generation studies shows that complex I deficiency cannot only be seen in MD but also in other genetic diseases especially if measured in muscle specimen of patients with terminal disease [51]. Hence, there is insufficient evidence that KD is more beneficial in mitochondrial complex I related disease than in other MD [11] or even other therapy refractory epilepsies. However, from our results we conclude that KD should be considered in MD patients with therapy resistant epilepsy.

Given the risk of AEs KD should be initiated by a team experienced with both MD and KD. Especially in the first weeks clinical and laboratory controls should be frequent (in line with the general guideline on KD). From our data we cannot conclude after which duration the efficacy can be judged and we therefore recommend a three months trial of KD, in analogy to KD for intractable epilepsy [52]. Whether classic KD or MAD is superior is unknown and an individual top-down (start KD 4:1) or bottom up approach (start with MAD) should be weighed and discussed with patient and/or parents.

### An appraisal for high fat diets

In this context, we would like to mention the high fat diets. The beneficial effects of KD for MDs are probably not only based on ketogenesis and energy expenditure from ketone bodies [22, 53]. Two studies that did not met our inclusion criteria, reporting 4 MD cases with complex I deficiency in muscle without a genetic diagnosis, showed improved maximal workload and muscle force under high fat diet [54, 55]. Another n = 1 trial reported a high fat diet improving the endurance in a bicycle test when compared to a high carbohydrate diet in one adult (*TMEM126B*) [50].

There is further interesting evidence to encourage human studies on high fat diets for MD First, supplementing of complex I deficient human fibroblast cell lines with palmitate resulted in protection from cell death caused by glucose withdrawal presumably based on fatty acid induced stimulation of mitochondrial biogenesis. Second, the study of a mouse model of Harlequin complex I deficient mice established that a high fat diet slowed down disease progression regarding major neurodegenerative symptoms and cerebellar atrophy [56].

More studies on high fat diet in humans reporting the aforementioned common data elements are necessary to draw conclusions. However, it also has to be considered that a high-fat diet could downregulate genes involved in the mitochondrial respiratory chain, and could thereby worsen the mitochondrial dysfunction [57].

## Conclusion

Data on efficacy and safety of KD for MD is too scarce for general recommendations. KD should be considered in individuals with MD and therapy refractory epilepsy, while mtDNA deletion(s) related myopathy is a contraindication (as well as Pyruvate Carboxylase deficiency). KD is a highly individual management option in this fragile patient group and requires an experienced team. To increase knowledge on this—individually—promising management option more (prospective) high quality studies using adequate outcome measures are crucial.

## Supplementary Information

**Additional file 1.** Search strategy.

**Additional file 2.** Excluded studies.

## Data Availability

All data generated or analysed during this study are included in this published article and its additional information files.

## Abbreviations

AE: Adverse event
KD: Ketogenic diet
MeSH: Medical subject headings
MD: Mitochondrial disease
MAD: Modified Atkins diet
OXPHOS: Oxidative phosphorylation
PRISMA: Preferred Reporting Items for Systematic Reviews and Meta-Analyses
PDHC: Pyruvate Dehydrogenase Complex
