# Correction to: Ketogenic diet for mitochondrial disease: a systematic review on efficacy and safety

**DOI:** 10.1186/s13023-021-02019-5

**Published:** 2021-09-27

**Authors:** Heidi Zweers, Annemiek M. J. van Wegberg, Mirian C. H. Janssen, Saskia B. Wortmann

**Affiliations:** 1 Department of Gastroenterology and Hepatology - Dietetics, Radboudumc, Postbus 9101, 6500 HB Nijmegen, The Netherlands; 2 Radboud Center for Mitochondrial Medicine (RCMM), Amalia Children’s Hospital, Radboudumc, Nijmegen, The Netherlands; 3 University Children’s Hospital, Paracelsus Medical University, Salzburg, Austria; 4 Department of Internal Medicine, Radboudumc, Nijmegen, The Netherlands

## Correction to: Orphanet J Rare Dis (2021) 16:295 10.1186/s13023-021-01927-w

Following the publication of the original article [1], the authors requested to move the declaration “SBW has the ERAPERMED2019-310 grant—Personalized Mitochondrial Medicine: Optimizing diagnostics and treatment for patients with mitochondrial diseases” from the ‘Competing interests’ section to the ‘Funding’ section of the article.

The ‘Funding’ section now states: “SBW has the ERAPERMED2019-310 grant—Personalized Mitochondrial Medicine: Optimizing diagnostics and treatment for patients with mitochondrial diseases”.

The ‘Competing interests’ section now states: “The authors declare that they have no competing interests”.

The ‘Funding’ and ‘Competing interests’ sections have already been updated in the original article.
