# Supplementary material for: Ketogenic diet for mitochondrial disease: a systematic review on efficacy and safety
Source: Orphanet J Rare Dis. 2021 Jul 3;16:295. doi: 10.1186/s13023-021-01927-w (PMC8254320; doi:10.1186/s13023-021-01927-w)
Supplement: Supplementary file 1 — Additional file 1. Search strategy. [file 13023_2021_1927_MOESM1_ESM.docx]

**Additional file 1 – Search strategy**

**Databases**
Four databases were searched
1. Pubmed
2. Cochrane
3. Embase (Ovid)
4. Cinahl

A combination was used of:
- Mesh terms (Medical Subject Headings) from Pubmed, Cochrane and Cinahl or Emtree terms from Embase
- Text-word search in title, abstract and author keywords.

**Entry terms**

1. "Mitochondrial Diseases"[Mesh]

2. alpers' OR alpers OR huttenlocher

3. complex deficienc*

4. complex 1 OR complex I OR complex 2 OR complex II OR complex 3 OR complex III OR complex 4 OR complex IV OR

complex 5 OR complex V

5. chronic progressive external ophthalmoplegia OR CPEO

6. [Friedreich Ataxia](https://www.ncbi.nlm.nih.gov/mesh/68005621)

7. Kearns Sayre

8. Leigh

9. Leber hereditary optic neuropathy OR LHON

10. mitochondrial encephalomyopathy lactic acidosis stroke-like episode* OR MELAS

11. myoclonic epilepsy ragged-red fibres OR MERRF

12. maternally inherited diabetes deafness OR MIDD

13. maternally inherited Leigh syndrome OR MILS

14. mitochondrial

15. [myoneurogenic gastrointestinal encephalopathy](https://en.wikipedia.org/wiki/Myoneurogenic_gastrointestinal_encephalopathy) OR MNGIE

16. mtDNA

17. [Neuropathy ataxia retinitis pigmentosa ptosis](https://en.wikipedia.org/wiki/Neuropathy,_ataxia,_and_retinitis_pigmentosa) OR NARP

18. Oxidative Phosphorylation Deficienc* OR OXPHOS

19. Pearson

20. POLG

21. Respiratory Chain Deficienc*

22. 1 or 2 or 3 or 4 or 6 or 6 or 7 or 8 or 9 or 10 or 11 or 11 or 12 or 13 or 14 or 15 or 16 or 17 or 18 or 19 or 20 or 21

23. "Diet, Ketogenic"[Mesh]

24. Ketogenic*

25. Modified Atkins

26. 23 or 24 or 25

27. 22 and 26

For Embase the following publications types were excluded: books or chapter or conference abstract or editorial or note or tombstone

**Syntax of Mesh/Emtree terms per database**

| **Entry nr** | **Pubmed** | **Cochrane** | **Embase (OVID)** | **Cinahl** |
| --- | --- | --- | --- | --- |
| 1 | "Mitochondrial Diseases"[Mesh] | MeSH descriptor: [Mitochondrial Diseases] explode all trees | exp "disorders of mitochondrial functions"/ | (MH "Mitochondrial Diseases+") |
| 23 | "Diet, Ketogenic"[Mesh] | MeSH descriptor: [Diet, Ketogenic] explode all trees | exp ketogenic diet/  exp modified Atkins diet/ | (MH "Ketogenic Diet") |

**Syntax of title, abstract and author keyword per database**

| **Entry nr** | **Pubmed** | **Cochrane** | **Embase (OVID)** | **Cinahl** |
| --- | --- | --- | --- | --- |
| 2-21, 24, 25 | [tiab] | :ti,ab,kw | .ti,ab,kw. | TI  AB |

Note:
High fat was left out as search term, as the focus is on ketogenic diet.
