# Supplementary material for: Ketogenic diet for mitochondrial disease: a systematic review on efficacy and safety
Source: Orphanet J Rare Dis. 2021 Jul 3;16:295. doi: 10.1186/s13023-021-01927-w (PMC8254320; doi:10.1186/s13023-021-01927-w)
Supplement: Supplementary file 2 — Additional file 2. Excluded studies. [file 13023_2021_1927_MOESM2_ESM.docx]

**Additional file 2 - Excluded studies**

| Study / Year | Reason for exclusion |
| --- | --- |
| Barzegar 2007 [1] | No genetic mitochondrial diagnose |
| Ait-El-Mkadem 2017[2] | Unknown diet composition |
| Al Madhoun 2019 [3] | Unknown diet composition |
| Banka, 2014[4] | Unknown diet composition |
| Bernard 2008 [5] | Wrong population: not on ketogenic diet |
| Bjorkman 2015 [6] | No clear or minimal outcome data |
| Brivet 2003 [7] | High fat diet |
| Cardenas 2010 [8] | Unknown diet composition |
| Emperador 2019 [9] | Wrong study type: cell study |
| Fahrner 2016 [10] | No genetic mitochondrial diagnose |
| Friederich 2020 [11] | No clear or minimal outcome data |
| Fukao 2019 [12] | Wrong population |
| Garcia-Cazorla 2005 [13] | No clear or minimal outcome data |
| Grazina 2007[14] | Unknown diet composition |
| Gropman 2018 [15] | Letter to the editor |
| Guilliams 2013 [16] | Letter to the editor |
| Han 2017 [17] | No clear or minimal outcome data |
| Habarou 2017[18] | Unknown diet composition |
| Hasan-Olive 2019 [19] | Wrong study type: animal study |
| Hildick-Smith 2013 [20] | No clear or minimal outcome data |
| Hussain 2016 [21] | Wrong population |
| Joshi 2016 [22] | No genetic mitochondrial diagnose |
| Jung 2012 [23] | No genetic mitochondrial diagnose |
| Kang 2006 [24] | No genetic mitochondrial diagnose |
| Kang 2007 [25] | No genetic mitochondrial diagnose |
| Khan 2012 [26] | Unknown diet composition |
| Kim 2012 [27] | No genetic mitochondrial diagnose |
| Klepper 2003 [28] | Not written in English |
| Klepper 2004 [29] | Not written in English |
| Kwong 2019 [30] | No genetic mitochondrial diagnose |
| Lee 2008 [31] | No genetic mitochondrial diagnose |
| Lee 2010 [32] | No genetic mitochondrial diagnose |
| Lee 2016 [33] | No clear or minimal outcome data |
| Lee 2019 [34] | No genetic mitochondrial diagnose |
| Leung 1998 [35] | No genetic mitochondrial diagnose |
| Liu 2019 [36] | Wrong population |
| Malojcic 2004 [37] | No genetic mitochondrial diagnose |
| Marchio 2016 [38] | abstract (conference published) |
| Martikainen 2012 [39] | Low glycemic index diet |
| Na 2020 [40] | No genetic mitochondrial diagnose |
| Nass 2019 [41] | No clear or minimal outcome data |
| Nathan 2019 [42] | Wrong population |
| Ngoh 2016 [43] | Unknown diet composition |
| Nishioka 2018 [44] | No clear or minimal outcome data |
| Nizon 2014 [45] | High fat diet |
| Nolan 2019 [46] | No clear or minimal outcome data |
| O'Connor 2014 [47] | No genetic mitochondrial diagnose |
| Oonthonpan 2019 [48] | Same case as Brivet 2003; high fat diet |
| Panetta 2004 [49] | Unknown diet composition |
| Pronicki 2017 [50] | Unknown diet composition |
| Punzi 2008 [51] | Unknown diet composition |
| Salman 2017 [52] | Wrong population: Glut 1 |
| Schmid 2019 [53] | No genetic mitochondrial diagnose |
| Seaver 2018 [54] | Unknown diet composition |
| Seo 2010 [55] | No genetic mitochondrial diagnose |
| Soler-Alfonso 2019 [56] | Unknown diet composition |
| Sort 2013 [57] | No genetic mitochondrial diagnose |
| Stowe 2018[58] | Unknown diet composition |
| Storoni 2019 [59] | Review, no human cases |
| Sutton 2010 [60] | Wrong population: secondary mitochondrial dysfunction |
| Taban 2006 [61] | Wrong population: PDHC deficiency |
| Theunissen 2017 [62] | High fat diet |
| Vasta 2012 [63] | No genetic mitochondrial diagnose |
| Villeneuve 2017 [64] | No genetic mitochondrial diagnose |
| Williams 2012 [65] | No clear or minimal outcome data |
| Yadav 2019 [66] | No genetic mitochondrial diagnose |
| Yamanaka 1987 [67] | No genetic mitochondrial diagnose |
| Yilmaz 2010 [68] | No genetic mitochondrial diagnose |
| Yoon 2014 [69] | No genetic mitochondrial diagnose |
| You 2009 [70] | No clear or minimal outcome data |
| Zhang 2018 [71] | No clear or minimal outcome data |
| Zupec-Kania 2013 [72] | Review, no human cases |

**References**

1. Barzegar, M. and M. Hashemilar, *Alpers disease: Report of two familial cases.* Pakistan Journal of Medical Sciences, 2007. **23**(4): p. 643-646.

2. Ait-El-Mkadem, S., et al., *Mutations in MDH2, Encoding a Krebs Cycle Enzyme, Cause Early-Onset Severe Encephalopathy.* Am J Hum Genet, 2017. **100**(1): p. 151-159.

3. Al Madhoun, A., et al., *Ketogenic diet attenuates cerebellar atrophy progression in a subject with a biallelic variant at the ATAD3A locus*. 2019. p. 79-86.

4. Banka, S., et al., *Expanding the clinical and molecular spectrum of thiamine pyrophosphokinase deficiency: A treatable neurological disorder caused by TPK1 mutations.* Molecular Genetics and Metabolism, 2014. **113**(4): p. 301-306.

5. Bernard, G. and M. Shevell, *The Wobbly Child: An Approach to Inherited Ataxias.* Seminars in Pediatric Neurology, 2008. **15**(4): p. 194-208.

6. Bjorkman, K., et al., *Broad phenotypic variability in patients with complex I deficiency due to mutations in NDUFS1 and NDUFV1.* Mitochondrion, 2015. **21**: p. 33-40.

7. Brivet, M., *Impaired mitochondrial pyruvate importation in a patient and a fetus at risk.* Molecular Genetics and Metabolism, 2003. **78**(3): p. 186-192.

8. Cardenas, J.F. and R.S. Amato, *Compound heterozygous polymerase gamma gene mutation in a patient with Alpers disease*. 2010. p. 62-4.

9. Emperador, S., et al., *Ketogenic treatment reduces the percentage of a LHON heteroplasmic mutation and increases mtDNA amount of a LHON homoplasmic mutation.* Orphanet J Rare Dis, 2019. **14**(1): p. 150.

10. Fahrner, J.A., et al., *A novel de novo dominant negative mutation in DNM1L impairs mitochondrial fission and presents as childhood epileptic encephalopathy.* Am J Med Genet A, 2016. **170**(8): p. 2002-11.

11. M.W., F., et al., *Pathogenic variants in NUBPL result in failure to assemble the matrix arm of complex I and cause a complex leukoencephalopathy with thalamic involvement.* Molecular Genetics and Metabolism, 2020. **129**(3): p. 236-242.

12. Fukao, T., et al., *Recent advances in understanding beta-ketothiolase (mitochondrial acetoacetyl-CoA thiolase, T2) deficiency.* Journal of Human Genetics, 2019. **64**(2): p. 99-111.

13. Garcia-Cazorla, A., et al., *Long-term follow-up of neonatal mitochondrial cytopathies: A study of 57 patients.* Pediatrics, 2005. **116**(5): p. 1170-1177.

14. Grazina, M.M., et al., *Atypical presentation of Leber's hereditary optic neuropathy associated to mtDNA 11778G>A point mutation--A case report.* Eur J Paediatr Neurol, 2007. **11**(2): p. 115-8.

15. Gropman, A. and A. Chiaramello, *Phenotypic spectrum of maternally inherited Leigh Syndrome associated with the m.8993T>G variant.* Molecular Genetics and Metabolism Reports, 2018. **15**: p. 134.

16. Guilliams, K., et al., *Hypothermia for pediatric refractory status epilepticus.* Epilepsia, 2013. **54**(9): p. 1586-1594.

17. Han, V.X., et al., *Novel LRPPRC Mutation in a Boy With Mild Leigh Syndrome, French-Canadian Type Outside of Quebec.* Child Neurol Open, 2017. **4**: p. 2329048X17737638.

18. Habarou, F., et al., *Biallelic Mutations in LIPT2 Cause a Mitochondrial Lipoylation Defect Associated with Severe Neonatal Encephalopathy.* Am J Hum Genet, 2017. **101**(2): p. 283-290.

19. Hasan-Olive, M.M., et al., *A Ketogenic Diet Improves Mitochondrial Biogenesis and Bioenergetics via the PGC1alpha-SIRT3-UCP2 Axis.* Neurochemical Research, 2019. **44**(1): p. 22-37.

20. Hildick-Smith, G.J., et al., *Macrocytic anemia and mitochondriopathy resulting from a defect in sideroflexin 4.* Am J Hum Genet, 2013. **93**(5): p. 906-14.

21. Hussain, K., T.J. Walsh, and J.L. Chazen, *Brain MRI findings with vigabatrin therapy: Case report and literature review.* Clinical Imaging, 2016. **40**(1): p. 180-182.

22. Joshi, C., et al., *Ketogenic diet - A novel treatment for early epileptic encephalopathy due to PIGA deficiency.* Brain and Development, 2016. **38**(9): p. 848-851.

23. Jung, D.E., et al., *Safety and role of ketogenic parenteral nutrition for intractable childhood epilepsy.* Brain and Development, 2012. **34**(8): p. 620-624.

24. Kang, H.C., et al., *Landau-Kleffner syndrome with mitochondrial respiratory chain-complex I deficiency.* Pediatr Neurol, 2006. **35**(2): p. 158-61.

25. Kang, H.C., et al., *Safe and effective use of the ketogenic diet in children with epilepsy and mitochondrial respiratory chain complex defects.* Epilepsia, 2007. **48**(1): p. 82-8.

26. Khan, A., et al., *Alpers syndrome: the natural history of a case highlighting neuroimaging, neuropathology, and fat metabolism.* J Child Neurol, 2012. **27**(5): p. 636-40.

27. Kim, Y.M., et al., *Various indications for a modified Atkins diet in intractable childhood epilepsy.* Brain Dev, 2012. **34**(7): p. 570-5.

28. Klepper, J. and B. Leiendecker, *Retogenic diet in disorders of cerebral energy metabolism.* Ernahrungs Umschau, 2003. **50**(12): p. 487-491.

29. Klepper, J., et al., *The ketogenic diet in German-speaking countries: Update 2003.* Klinische Padiatrie, 2004. **216**(5): p. 277-285.

30. Kwong, A.K., et al., *ARX-associated infantile epileptic-dyskinetic encephalopathy with responsiveness to valproate for controlling seizures and reduced activity of muscle mitochondrial complex IV.* Brain Dev, 2019. **41**(10): p. 883-887.

31. Lee, Y.M., et al., *Mitochondrial respiratory chain defects: underlying etiology in various epileptic conditions*. Vol. 49. 2008. 685-90.

32. Lee, S.-K., et al., *Initial experiences with proton MR spectroscopy in treatment monitoring of mitochondrial encephalopathy.* Yonsei medical journal, 2010. **51**(5): p. 672-5.

33. Lee, H.N., et al., *Epilepsy Characteristics and Clinical Outcome in Patients With Mitochondrial Encephalomyopathy, Lactic Acidosis, and Stroke-Like Episodes (MELAS).* Pediatric Neurology, 2016. **64**: p. 59-65.

34. Lee, S., M.S. Baek, and Y.M. Lee, *Lennox-Gastaut Syndrome in Mitochondrial Disease.* Yonsei Med J, 2019. **60**(1): p. 106-114.

35. Leung, T.F., et al., *A Chinese girl with Leigh syndrome: effect of botulinum toxin on dystonia.* Journal of paediatrics and child health, 1998. **34**(5): p. 480-2.

36. Liu, H., et al., *Severe clinical manifestation of mitochondrial 3-hydroxy-3-methylglutaryl-CoA synthase deficiency associated with two novel mutations: a case report.* BMC pediatrics, 2019. **19**(1): p. 344.

37. Malojcic, B., et al., *An adult case of Leigh disease.* Clinical neurology and neurosurgery, 2004. **106**(3): p. 237-40.

38. Marchio, M., et al., *Application of the ketogenic diet in inherited metabolic diseases.* 2016: p. S93.

39. Martikainen, M.H., et al., *Successful treatment of POLG-related mitochondrial epilepsy with antiepileptic drugs and low glycaemic index diet.* Epileptic Disord, 2012. **14**(4): p. 438-41.

40. Na, J.H., H.D. Kim, and Y.M. Lee, *Effective and safe diet therapies for Lennox-Gastaut syndrome with mitochondrial dysfunction.* Ther Adv Neurol Disord, 2020. **13**: p. 1756286419897813.

41. Nass, R.D., et al., *Retinoencephalopathy with occipital lobe epilepsy in an OPA-1 mutation carrier.* Seizure, 2019. **66**: p. 1-3.

42. Nathan, J., et al., *Substantial Remission in Subacute Sclerosing Panencephalitis by Following the Ketogenic Diet: A Case Report*. 2019. p. e5485.

43. Ngoh, A., et al., *RARS2 mutations in a sibship with infantile spasms.* Epilepsia, 2016. **57**(5): p. e97-e102.

44. Nishioka, M., et al., *An infant case of diffuse cerebrospinal lesions and cardiomyopathy caused by a BOLA3 mutation*. 2018. p. 484-488.

45. Nizon, M., et al., *Leukoencephalopathy with cysts and hyperglycinemia may result from NFU1 deficiency.* Mitochondrion, 2014. **15**: p. 59-64.

46. Nolan, D.A., et al., *A Rasmussen encephalitis, autoimmune encephalitis, and mitochondrial disease mimicker: expanding the DNM1L-associated intractable epilepsy and encephalopathy phenotype*. 2019. p. 112-116.

47. O'Connor, S.E., et al., *The ketogenic diet for the treatment of pediatric status epilepticus.* Pediatr Neurol, 2014. **50**(1): p. 101-3.

48. Oonthonpan, L., et al., *Two human patient mitochondrial pyruvate carrier mutations reveal distinct molecular mechanisms of dysfunction.* JCI Insight, 2019. **4**(13): p. e126132.

49. Panetta, J., L.J. Smith, and A. Boneh, *Effect of high-dose vitamins, coenzyme Q and high-fat diet in paediatric patients with mitochondrial diseases.* J Inherit Metab Dis, 2004. **27**(4): p. 487-98.

50. Pronicki, M., et al., *Neuropathological characteristics of the brain in two patients with SLC19A3 mutations related to the biotin-thiamine-responsive basal ganglia disease.* Folia Neuropathol, 2017. **55**(2): p. 146-153.

51. Punzi, G., et al., *SLC25A10 biallelic mutations in intractable epileptic encephalopathy with complex I deficiency.* Hum Mol Genet, 2018. **27**(3): p. 499-504.

52. Salman, M.S., S.F. Klassen, and J.L. Johnston, *Recurrent Ataxia in Children and Adolescents.* Canadian Journal of Neurological Sciences, 2017. **44**(4): p. 375-383.

53. Schmid, S.J., et al., *A de Novo Dominant Negative Mutation in DNM1L Causes Sudden Onset Status Epilepticus with Subsequent Epileptic Encephalopathy.* Neuropediatrics, 2019. **50**(3): p. 197-201.

54. Seaver, L.H., et al., *Lethal NARS2-Related Disorder Associated With Rapidly Progressive Intractable Epilepsy and Global Brain Atrophy.* Pediatr Neurol, 2018. **89**: p. 26-30.

55. Seo, J.H., et al., *A case of Ohtahara syndrome with mitochondrial respiratory chain complex I deficiency.* Brain & development, 2010. **32**(3): p. 253-7.

56. Soler-Alfonso, C., et al., *L-Cysteine supplementation prevents liver transplantation in a patient with TRMU deficiency.* Mol Genet Metab Rep, 2019. **19**: p. 100453.

57. Sort, R., et al., *Ketogenic diet in 3 cases of childhood refractory status epilepticus*. Vol. 17. 2013. 531-6.

58. Stowe, R.C., et al., *LIPT1 deficiency presenting as early infantile epileptic encephalopathy, Leigh disease, and secondary pyruvate dehydrogenase complex deficiency*. 2018. p. 1184-1189.

59. Storoni, M., M.P. Robert, and G.T. Plant, *The therapeutic potential of a calorie-restricted ketogenic diet for the management of Leber hereditary optic neuropathy.* Nutritional Neuroscience, 2019. **22**(3): p. 156-164.

60. Sutton, M.B., et al., *Resilience, childhood disability and the Internet - Case report.* Wiener Medizinische Wochenschrift, 2010. **160**(13-14): p. 325-327.

61. Taban, M., et al., *Association of optic nerve hypoplasia with mitochindrial cytopathies.* Journal of Child Neurology, 2006. **21**(11): p. 956-960.

62. Theunissen, T.E.J., et al., *Selection and Characterization of Palmitic Acid Responsive Patients with an OXPHOS Complex I Defect.* Front Mol Neurosci, 2017. **10**: p. 336.

63. Vasta, V., et al., *Next-generation sequencing for mitochondrial diseases: A wide diagnostic spectrum.* Pediatrics International, 2012. **54**(5): p. 585-601.

64. Villeneuve, N., et al., *Heterogeneity of FHF1 related phenotype: Novel case with early onset severe attacks of apnea, partial mitochondrial respiratory chain complex II deficiency, neonatal onset seizures without neurodegeneration.* Eur J Paediatr Neurol, 2017. **21**(5): p. 783-786.

65. Williams, E., et al., *A parent's perspective on dietary treatments for epilepsy.* Epilepsy Research, 2012. **100**(3): p. 338-343.

66. Yadav, P. and S. Variar, *Leigh syndrome: Case report and review of literature.* Journal of Clinical and Diagnostic Research, 2019. **13**(12): p. TD01-TD03.

67. Yamanaka, R., et al., *MELAS, myoclonus, ataxia and deficiencies of complexes I and IV in muscle mitochondria.* Acta Paediatrica Japonica (Overseas Edition), 1987. **29**(5): p. 761-767.

68. Yilmaz, A.A., et al., *Respiratory failure in Leigh syndrome.* Anestezi Dergisi, 2010. **18**(4): p. 225-227.

69. Yoon, J.R., et al., *Polyunsaturated fatty acid-enriched diet therapy for a child with epilepsy.* Brain Dev, 2014. **36**(2): p. 163-6.

70. You, S.J., H.D. Kim, and H.C. Kang, *Factors Influencing the Evolution of West Syndrome to Lennox-Gastaut Syndrome.* Pediatric Neurology, 2009. **41**(2): p. 111-113.

71. Zhang, J., et al., *Distinct magnetic resonance imaging features in a patient with novel RARS2 mutations: A case report and review of the literature.* Exp Ther Med, 2018. **15**(1): p. 1099-1104.

72. Zupec-Kania, B., et al., *An update on diets in clinical practice.* Journal of Child Neurology, 2013. **28**(8): p. 1015-1026.
